# Host-Parasite Interactions in Individuals with Type 1 and 2 Diabetes Result in Higher Frequency of *Ascaris lumbricoides* and *Giardia lamblia* in Type 2 Diabetic Individuals

**DOI:** 10.1155/2018/4238435

**Published:** 2018-02-06

**Authors:** Eleuza Rodrigues Machado, Núbia Oliveira Matos, Sinione Morais Rezende, Daniela Carlos, Thauana Cristina Silva, Leônia Rodrigues, Maria Jarlene Rodrigues Almeida, Maria Regina Fernandes de Oliveira, Maria Imaculada Muniz-Junqueira, Rodrigo Gurgel-Gonçalves

**Affiliations:** ^1^Curso de Enfermagem, Faculdade Anhanguera de Brasília, Unidade Taguatinga, Universidade Kroton, Taguatinga, DF, Brazil; ^2^Laboratório de Parasitologia Médica e Biologia de Vetores, Área de Patologia, Faculdade de Medicina, Universidade de Brasília, Asa Norte, 70904-970 Brasilia, DF, Brazil; ^3^Laboratório de Imunologia, Departamento de Bioquímica e Imunologia, Faculdade de Medicina, Universidade de São Paulo de Ribeirão Preto, Ribeirão Preto, SP, Brazil; ^4^Núcleo de Medicina Tropical, Faculdade de Medicina, Universidade de Brasília, Brasilia, DF, Brazil; ^5^National Institute for Science and Technology for Health Technology Assessment (IATS/CNPq), Porto Alegre, RS, Brazil; ^6^Laboratório de Imunologia Celular, Área de Patologia, Faculdade de Medicina, Universidade de Brasília, Brasilia, DF, Brazil

## Abstract

Host-parasite interactions in diabetic patients might influence diabetes complications and intestinal parasitosis. The aim was to investigate the occurrence of enteroparasites in individuals with diabetes types 1 and 2. A descriptive study was designed to estimate frequencies of parasites and to compare them in individuals with diabetes types 1 and 2 from two Health Centers and one hospital in the Federal District of Brazil. Patients were allocated to the study by convenience. Three fecal samples of 156 diabetic individuals (120 type 1 and 36 type 2) were analyzed using two parasitological methods. Enteroparasites or commensals frequency in diabetics was 64%. Diabetics infected with up to six species of intestinal parasites or commensals were found. Frequencies of *Ascaris lumbricoides* and *Giardia lamblia* were higher in individuals with type 2 diabetes. The lower frequency of *A. lumbricoides* found in type 1 diabetes may be related to a strong Th2 response to parasites. Autoimmune response developed in type 1 diabetic individuals characterized by the production of Th1 cytokines could explain low frequency of *G. lamblia*. High frequency of parasites found in type 2 diabetes emphasizes the importance of periodic parasitological examinations in these individuals.

## 1. Introduction

Intestinal parasites occur in marginalized, low-income, and resource-constrained regions of the world, with over one billion infected people in developing areas of Africa, Asia, and the Americas [1]. Diabetes affects more than 300 million individuals globally, contributing to significant morbidity and mortality worldwide [2], and more than 80% of diabetes deaths occur in low- and middle-income countries [3].

There are immunological interactions between helminths and their hosts, in which helminths polarize the immune system towards a strong type-2 immune response that is associated with immune defense and tissue repair. In addition, the establishment of a regulatory network can contribute to the control of overt immune responses to allow longer survival of the parasite while restricting inflammation that might otherwise lead to pathology [4]. These alterations in the host immune state might influence and be influenced by other concomitant diseases [5].

Type 1 diabetes (T1D) arises following the autoimmune destruction of insulin-producing pancreatic *β* cells. The development of diabetes has been linked to the loss of self-tolerance to beta cell autoantigens leading to the Th1-mediated destruction of insulin-producing beta cells. Helminths might inhibit type1 diabetes by disrupting the pathways leading to the Th1-mediated destruction of insulin-producing beta cells mediated by mechanisms related to the capacity of the host to mount a Th2 response to parasites. T1D accounts for 90% of diabetes in children and adolescents, usually presenting with a classic trio of symptoms such as polydypsia, polyphagia, and polyuria, alongside of overt hyperglycemia [6].

Type 2 diabetes (T2D) is regarded as an inflammatory disease lacking specific disease-related antigens. There is an association between chronic inflammation in adipose tissue and the development of insulin resistance and T2D, a condition associated to the metabolic syndrome [7]. There are robust inflammatory cell infiltration and M1 type macrophages in the islets of patients with type 2 diabetes [8]. This form of diabetes, which accounts for 90–95% of those with diabetes, encompasses mainly adult individuals [9].

There is a large spatial overlap between intestinal parasites and diabetes distribution, and the pathogenic mechanisms of both diseases suggest that they might influence each other; however, few studies on the occurrence of intestinal parasites in diabetic individuals were made until now [4]. It has been suggested that parasite infections might influence diabetes frequency, both by decreasing or increasing it. Experimental data suggest that exposure to helminth infections can inhibit the development of chronic inflammatory diseases, such as T1D and other immune-mediated diseases [10]. Otherwise, clinical trials showed that intestinal parasite prevalence in the diabetic group was significantly lower than in the control subject group [11], and Hakim et al. [12] showed higher prevalence giardiasis in diabetic patients (15%, mean age 54 years) than in dyspeptic individuals (7%), but no discrimination between T1D and T2D groups was done in these papers. Individuals who had previously *S. stercoralis* infection were 61% less likely to have a diagnosis of T2D than those uninfected [13]. Whereas opposite results were also observed by Mendonça et al. [14]; they observed that positive *S. stercoralis* serology in diabetics is significantly more frequent in T2D patients (23%) than control individuals (7.1%).

Considering the high frequency of intestinal parasites and the increasing number of diabetics [15], it is relevant to know the rates of infection of diabetic individuals by protozoa and helminths for the development of treatment strategies for these individuals. Furthermore, as immunological and physiopathological mechanisms causing T1D and T2D are different, the aim of this study was to investigate the occurrence of intestinal parasites in individuals with T1D and T2D.

## 2. Materials and Methods

A descriptive study was designed to estimate frequencies of parasites and to compare them in individuals with T1D and T2D.

### 2.1. Population

Diabetic patients from two health centers and one hospital located in Taguatinga, Federal District, Brazil, were evaluated. The patients were allocated to the study by convenience. In total, 125 patients from the hospital (117 T1D and 8 T2D) and 31 from health centers (28 T2D and 3 T1D) were included. Outpatients were seen at an internal medicine ambulatory clinic. Patients were from a group of diabetic individuals attending lectures on diabetes topics every 15 days. The research project was presented in these meetings. Afterwards, the patients were contacted by phone and the scheduling was done for orientation on the stool collection and signing of the informed consent. During the orientation, they received the stool collectors and the delivery dates of the stool samples in the hospital or health centers were scheduled.

The clinical signs were considered to differentiate between the T1D and T2D. The characteristics used for the diagnosis of the T1D were appearance of the disease in the juvenile phase, being thin or losing weight with little or no presence of endogenous insulin, having anti-insulin autoantibodies, and the need of exogenous insulin; the patients were prone to ketosis in the absence of insulin, and in the acute phase of the hyperglycemia, they might exhibit diabetic ketoacidosis. On the other hand, the characteristic symptoms of T2D were onset of illness in the adult phase, if the patients are not dependent on insulin, having no autoantibodies, and if the origin of the disease were associated with obesity, heredity, and environmental factors. Inclusion criteria were (i) fasting plasma glucose (FPG) ≥ 126 mg/dl or symptoms (such as polyuria, polydipsia, and unexplained weight loss), (ii) random plasma glucose ≥200 mg/dL^9^, and (iii) three fecal samples obtained from each individual. Patients treated with antiparasite drugs in the past 6 months, with any other clinically significant intestinal disease, allergies, or those who presented diseases that authors judged might influence the presence of intestinal parasitism, were excluded.

### 2.2. Sampling

Based on general prevalence estimated by previous study [16], the sample was estimated, using one-tailed sampling for two independent groups (G^∗^Power v3.1.9.2). The sampling parameters were P1 = 0.54, P2 = 0.30, *α* = 0.05, sample power = 0.80, and ratio N2/N1 = 3. The final estimated number was 156, with 39 individuals in T2D group and 117 in T1D group.

### 2.3. Ethical Aspects

The objectives of the study were presented to patients or their guardians and they signed the informed consent. Diabetes disease was followed by the medical staff, and all infected individuals were treated by the end of the work. This study was approved by the Ethics Committee of Secretariat of Health of FD (project number 172/2011-CEP/SES/DF).

### 2.4. Parasitological Analysis

Three fecal samples from each individual were collected from July 2011 to October 2012. Fecal samples were collected using universal collectors, without preservatives, at intervals of three days between samples. The vials were labeled, stored in a polystyrene box, and sent to the Laboratory of Medical Parasitology and Vector Biology, University of Brasilia where they were analyzed using two fecal parasitological methods [17, 18]. Nine slides were prepared for each of the three fecal samples, and the samples were blindly evaluated by three investigators. The diagnostic procedure was performed as described elsewhere [19].

### 2.5. Data Analysis

The occurrence of intestinal parasites among individuals with T1D or T2D was analyzed using descriptive statistics, chi-square, or exact Fisher's tests (when the expected values in any of the cells of a contingency table were below 5), to check for differences in the proportion of T1D or T2D individuals infected or not infected by the species of intestinal parasites or commensals diagnosed. Statistical tests were performed using Statistica® and considering *p* < 0.01 as statistically significant. Proportions and confidence intervals (lower and upper) of T1D or T2D individuals infected by the species of protozoan and/or helminths were also estimated. The proportions and confidence intervals were estimated using the Agresti and Coull's method [20].

## 3. Results

In total, 120 individuals with T1D and 36 with T2D were included from the three selected health services. Among T1D individuals, 68 (57%) were female and 87 (73%) were aged from one to 30 years old. Among T2D individuals, 28 (78%) were female and 22 (61%) had ages ranging from 51 to 70 years old (Table 1). In total, 118 T1D individuals (98%) and 13 T2D (36%) were in regular use of insulin. For each individual, 27 samples were analyzed totaling 4617 observations. The overall frequency of intestinal parasites in diabetic individuals was 64%. *Entamoeba coli* (42%), *Endolimax nana* (23%), *Giardia lamblia* (16%), and *Entamoeba hartmanni* (10%) were the main protozoa detected. *Ascaris lumbricoides* (12%), *Taenia* sp. (3%), hookworms (2%), *Hymenolepis nana* (1%), *Strongyloides stercoralis* (1%), *Enterobius vermicularis* (0.6%), and *Schistosoma mansoni* (0.6%) were the helminths detected.

**Figure 1:**
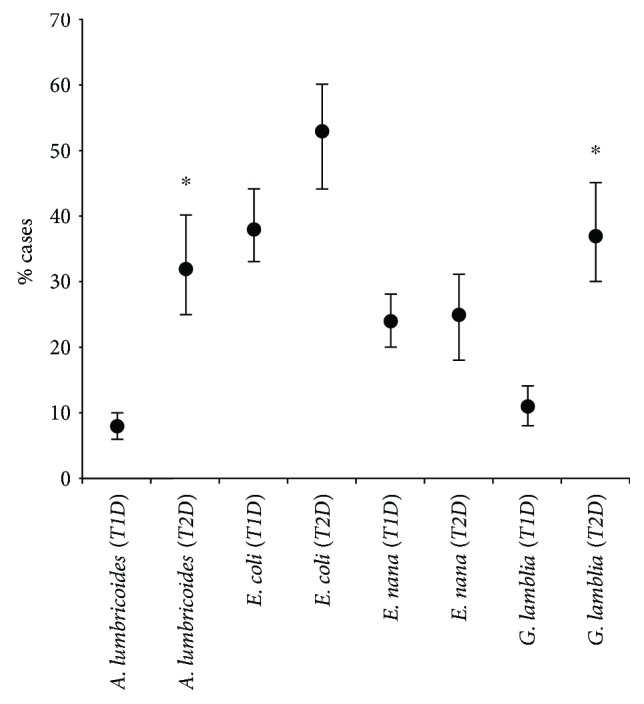
Estimated proportions and confidence intervals (lower and upper) of T1D and T2D patients infected with the most frequent parasites and commensals detected in the study, Federal District of Brazil, between 2011 and 2012. ^∗^*p* < 0.01 as statistically significant difference.

Among individuals showing T1D, 62% (74/120) were infected with protozoa and/or helminths, whereas 78% (28/36) of that showing T2D were infected. *G. lamblia* and *A. lumbricoides* were the most common parasite species found in T2D patients and individuals infected with up to six species of intestinal parasites or commensals were found (Table 2). All 468 samples analyzed by the method of Rugai were negative. Significant differences were detected between the infection rate of T1D and T2D individuals for *G. lamblia* (*p* < 0.01) and for *A. lumbricoides* (*p* < 0.01). The frequencies of *A. lumbricoides* and *G. lamblia* were higher in individuals with type 2 diabetes (Figure 1).

## 4. Discussion

The present study showed that patients with T1D were significantly less parasitized with intestinal *G. lamblia* and *A. lumbricoides* than those with T2D. Helminth infections might protect against T1D diabetes development by disrupting the pathways leading to the Th1-mediated destruction of insulin-producing beta cells mediated by mechanisms related to the capacity of the host to mount a Th2 response to parasites, thus, decreasing the frequency of T1D [4]. Potent type 2 immune response is triggered by helminths that might inhibit islet beta cell-specific interferon gamma (IFN-g) producing Th1 cells and might increase interleukin- (IL-) 4, transforming growth factor beta (TGF-b), and autoantigen-specific T cells producing IL-10 [21]. The fact that parasites have been observed in children since nine months old reinforces this possibility [22]. In addition, it should be considered that the autoimmune response developed in patients with T1D, characterized by the production of Th1 cytokines such as IL-2, TNF-*α*, IFN-*γ*, and T CD8^+^ cytotoxic lymphocytes in association with T CD4^+^ lymphocytes [10], could eliminate *G. lamblia* [23]. Some studies show that individuals with giardiasis have TNF-*α* and IL-2 high, similar to Th1 response [24]. This fact could also support the lower *G. lamblia* infection in individuals with T1D.

The explanation for higher frequency of TD2 individuals with *A. lumbricoides* or *G. lamblia* is less evident. It must be considered that the higher range of age of these individuals suggests that the parasite infection might occur years before the development of the T2D metabolic disease. Then, when T2D occurs, the strong switch to Th2 response caused by parasites has already decreased. Thus, the proinflammatory immune response related to the metabolic disturb has prevailed. Then, the onset of *A. lumbricoides* or *G. lamblia* infection, before or after the development of diabetes, should be considered to understand the pathophysiological mechanisms of the disease. Hyperglycemia and dyslipidemia activate proinflammatory mediators through the involvement of several metabolic pathways. Once these proinflammatory mediators are released, they induce tissue-specific inflammation to which insulin resistance in peripheral tissues and impaired insulin secretion in pancreatic islets occur that ultimately enhance the chance of development of the T2D [25] and decrease the immune defense against parasites.

These factors could explain the lower frequency of *A. lumbricoides* in T1D individuals, even considering the lower age range of this group of individuals (mostly children and young adults), which would be epidemiologically more susceptible than T2D (mostly old adults) to infections by intestinal parasites.

The present study did not evaluate the prevalence of parasites in nondiabetic individuals, a limitation that could be addressed in future studies comparing diabetic (T1D and T2D) and nondiabetic individuals. Moreover, immunological profiles of T1D and T2D parasite-infected individuals may help in understanding the factors associated with intestinal parasite occurrence in diabetic individuals.

## 5. Conclusions

In conclusion, our study showed lower frequency of *A. lumbricoides* and *G. lamblia* in T1D; this may be related to host immune response. High frequency of protozoan and helminths was found in T2D and emphasizes the importance of periodic parasitological examinations in these individuals to allow rapid treatment and prevent severe infections.

## Figures and Tables

**Table 1:** Distribution of individuals with type 1 diabetes (T1D, *n* = 120) and type 2 (T2D, *n* = 36) included in the study according to age and sex. Individuals were examined in health centers and the regional hospital of Taguatinga, Federal District, Brazil, 2011-2012.

Age (years)	Sex		Diabetes	
	Males	Females	T1D	T2D
<10	13	9	22	0
11 to 20	13	27	40	0
21 to 30	11	14	25	0
31 to 40	9	12	19	2
41 to 50	2	8	6	4
51 to 60	3	13	6	10
61 to 70	7	6	1	12
71 to 80	2	6	1	7
>81	0	1	0	1

**Table 2:** Frequency of intestinal protozoa and helminths detected in fecal samples of individuals with type 1 diabetes (T1D, *n* = 120) and type 2 (T2D, *n* = 36), examined in health centers and the regional hospital of Taguatinga, Federal District, Brazil, 2011-2012.

Parasites species	Diabetic individuals *n* (%)		*p* ^∗^
	T1D	T2D	
Protozoans			
*Giardia lamblia*	12 (10)	13 (36)	<0.01
*Entamoeba histolytica/dispar*	1 (0.8)	1 (3)	0.40
*Balantidium coli*	0 (0)	1 (3)	0.40
Helminths			
*Ascaris lumbricoides*	8 (7)	11 (30)	<0.01
*Taenia* sp.	4 (3)	1 (3)	1.00
Hookworms	1 (0.8)	2 (6)	0.13
*Hymenolepis nana*	1 (0.8)	1 (3)	0.40
*Strongyloides stercoralis*	1 (0.8)	0 (0)	1.00
*Enterobius vermicularis*	1 (0.8)	0 (0)	1.00
*Schistosoma mansoni*	1 (0.8)	0 (0)	1.00
Commensals			
*Entamoeba coli*	46 (38)	19 (53)	0.12
*Endolimax nana*	28 (23)	8 (22)	0.89
*Entamoeba hartmanni*	15 (12)	1 (3)	1.00
Multiple infections			
*E. coli + E. nana*	8 (7)	1 (3)	0.69
*E. coli + E. hartmanni*	5 (4)	0 (0)	0.59
*E. coli + G. lamblia*	3 (2.5)	4 (11)	0.05
*E. coli + A. lumbricoides*	2 (2)	4 (11)	0.03
*E. hartmanni + G. lamblia*	3 (2.5)	0 (0)	1.00
*E. coli +* hookworms	1 (0.8)	0 (0)	1.00
*E. hartmanni + E. nana*	1 (0.8)	0 (0)	1.00
*E. coli + Taenia* sp.	1 (0.8)	0 (0)	1.00
*E. hartmanni + A. lumbricoides*	1 (0.8)	0 (0)	1.00
*G. lamblia + A. lumbricoides*	1 (0.8)	2 (6)	0.13
*E. nana + G. lamblia*	0 (0)	1 (3)	0.23
*E. coli + S. stercoralis*	0 (0)	1 (3)	0.23
*E. coli + E. nana + E. hartmanni*	3 (2.5)	1 (3)	1.00
*E. coli + G. lamblia +* hookworms	0 (0)	1 (3)	0.23
*E. coli + G. lamblia + S. stercoralis*	0 (0)	1 (3)	0.23
*E. coli + E. nana + G. lamblia*	0 (0)	1 (3)	0.23
*E. coli + G. lamblia + A. lumbricoides*	0 (0)	1 (3)	0.23
*E. coli + G. lamblia + A. lumbricoides + Taenia* sp*. +* hookworms	0 (0)	1 (3)	0.23
*E. coli + E. nana + G. lamblia + Taenia* sp. *+* hookworm *+ S. stercoralis*	0 (0)	1 (3)	0.23

^∗^Chi-square or exact Fisher's tests (when the expected values in any of the contingency table cells were below 5).
